# Determinants of nonsuppression of HIV viral load among children receiving antiretroviral therapy in the Simiyu region: a cross-sectional study

**DOI:** 10.1186/s12981-023-00515-1

**Published:** 2023-04-13

**Authors:** Kihulya Mageda, Khamis Kulemba, Wilhelmina Olomi, Ntuli Kapologwe, Leornad Katalambula, Pammla Petrucka

**Affiliations:** 1 School of Nursing and Public Health, University of Dodoma, PO Box 395, Dodoma, Tanzania; 2 Department of Health, Simiyu Regional Commissioners’ Office, Bariadi, Tanzania; 3 National Institute for Medical Research Mbeya, Mbeya, Tanzania; 4 University of Saskatchewan, Saskatoon, Canada; 5 President’s Office–Regional Administration and Local Government, PO Box 1923, Dodoma, Tanzania

**Keywords:** Viral load suppression, HIV-infected children, Antiretroviral therapy, Adherence

## Abstract

**Background:**

Despite substantial antiretroviral therapy (ART) coverage among individuals with human immunodeficiency virus (HIV) infection in Tanzania, viral load suppression (VLS) among HIV-positive children receiving ART remains intolerably low. This study was conducted to determine factors affecting the nonsuppression of VL in children with HIV receiving ART in the Simiyu region; thus, an effective, sustainable intervention to address VL nonsuppression can be developed in the future.

**Methods:**

We conducted a cross-sectional study including children with HIV aged 2–14 years who were currently presenting to care and treatment clinics in the Simiyu region. We collected data from the children/caregivers and care and treatment center databases. We used Stata™ to perform data analysis. We used statistics, including means, standard deviations, medians, interquartile ranges (IQRs), frequencies, and percentages, to describe the data. We performed forward stepwise logistic regression, where the significance level for removal was 0.10 and that for entry was 0.05. The median age of the patients at ART initiation was 2.0 years (IQR, 1.0–5.0 years), and the mean age at HIV VL (HVL) nonsuppression was 8.8 ± 2.99 years. Of the 253 patients, 56% were female, and the mean ART duration was 64 ± 33.07 months. In multivariable analysis, independent predictors of HVL nonsuppression were older age at ART initiation (adjusted odds ratio [AOR] = 1.21; 95% confidence interval [CI] 1.012–1.443) and poor medication adherence (AOR, 0.06; 95% CI 0.004–0.867).

**Conclusions:**

This study showed that older age at ART initiation and poor medication adherence play significant roles in HVL nonsuppression. HIV/AIDS programs should have intensive interventions targeting early identification, ART initiation, and adherence intensification.

## Background

The introduction of antiretroviral therapy (ART) and increased access to effective human immunodeficiency virus (HIV) prevention, diagnosis, treatment, and care, including opportunistic infection prevention, have heralded essential changes in the natural course of HIV infection in adults and children [1, 2]. Therefore, morbidity and mortality in adults and children have decreased [3].

To end the AIDS epidemic as a public health risk, the Joint United Nations Programme on HIV/AIDS (UNAIDS) Fast-Track approach set a target for 95% of all people living with HIV (PLHIV) to know their HIV status (diagnosed). In addition to this target, corresponding targets include the following: 95% of people with HIV infection should be started on ART, 95% of those on ART should achieve sustainable viral suppression by 2030, and there should be zero discrimination [4]. The attainment of these targets will potentially reduce new HIV infections by 95%, thus providing an opportunity to end the AIDS epidemic by 2030 [5].

Viral load suppression (VLS) is an essential parameter of ART effectiveness as a surrogate marker for disease progression [6, 7]. The aim of ART administration in children is to suppress HIV replication and halt disease progression while reducing opportunistic infections and morbidity [8, 9]. Subsequently, this approach promotes optimal growth and maximizes the health and well-being of children with HIV infection. Unfortunately, a low proportion of children with HIV in the Eastern Africa region compared with those in other countries in sub-Saharan Africa have a high risk of developing AIDS [10, 11]. In Tanzania, National AIDS Control Program data and the Tanzania HIV Impact Survey show that VLS in pediatric patients continues to be low [12–14]. Several studies have reported various factors causing VL nonsuppression, such as poor adherence, comorbidities [15–17], malnutrition [18, 19], underlying tuberculosis infection [20], and advanced disease [21].

In Tanzania, most HIV infections in children are acquired through mother-to-child transmission during pregnancy, delivery, or breastfeeding [22]. In children older than 18 months, the diagnosis of HIV infection is made through the detection of antibodies using HIV rapid tests. In infants and children under the age of 18 months, HIV infection is confirmed by HIV DNA polymerase chain reaction. HVL monitoring is performed routinely to monitor children, usually 6 months post-ART initiation. If the VL is more significant than 1000 copies/mL, the caregiver receives enhanced adherence counseling and the child is retested after 3 months [22].

Despite substantial ART coverage in PLHIV in Tanzania, VLS among HIV-positive children receiving ART remains intolerably low at 18%, indicating that 82% of those enrolled at care and treatment centers (CTCs) receiving ART do not have VLS [12]. Despite the efforts made by the Tanzania National HIV Control Program to increase retention and adherence to care, VLS has not been effectively achieved among children [23].

Therefore, this study was designed to determine factors affecting VL nonsuppression in children with HIV infection receiving ART. The findings of this study will help us fill the knowledge gap about the factors contributing to VL nonsuppression among children with HIV infection in Tanzania.

## Methods

### Study design and setting

We conducted a cross-sectional analytical study in the Simiyu region. Administratively, the region comprises six districts with an estimated population of 2 million individuals. It has 218 health facilities (8 hospitals, 17 health centers, and 193 dispensaries), of which 106 provide ART services [24]. We obtained our sample from 48 of the 106 facilities. The main activity of the population in this region is agriculture, followed by livestock care. We conducted this study from October 25 to November 30, 2021.

### Study population

The study population included children with HIV aged 2–14 years who were enrolled or currently receiving treatment at CTCs.

### Sample size determination

We calculated the required sample size using the following formula [25]:

$$\text{n = }{{\text{Z}}^{\text{2}}}\text{P }\left( 1-\text{P} \right)\text{/}{{\varepsilon}^{\text{2}}},$$

where n is the minimum sample size; Z is the standard average deviation of 1.96, corresponding to a 95% confidence interval (CI); P is the prevalence of VLS in children, which was 18% [26]; and ε indicates the degree of accuracy of the results, which was 0.05.

The calculated final sample size, with an additional 10% for incomplete data, was 253.

### Sampling procedure

Multistage stratified sampling was used to select CTCs for inclusion in the study. Of the 106 health facilities offering care and treatment services (i.e., CTCs), five were hospitals, 16 were health centers, and 85 were dispensaries. All hospitals and health centers were included in the study, whereas one-third of the dispensaries were randomly selected from each district for inclusion. During the study, one health center was excluded because two health centers were present in the same location, so random selection was performed to include one health center. A total of 48 facilities were included to represent CTCs in the Simiyu region, including 28 dispensaries, 15 health centers and five hospitals. All children with HIV infection aged between 2 and 14 years who presented to the selected health facilities and met the eligibility criteria were included in the study.

### Data collection

The study was introduced to all caregivers with their children in waiting rooms, and the children were screened for inclusion criteria after receiving routine care. After obtaining written informed consent, the caregivers were interviewed using a questionnaire adapted from the National AIDS Control Program to collect basic demographic information, such as the current age and educational status of the child, the caregiver’s relationship to the child, source of income, and distance from the home to the health facility. Other child clinical and demographic information, such as the child’s age at ART initiation, sex, ART duration, regimen, weight, WHO clinical stage, ART adherence, and viral load, were extracted from the CTC electronic databases and exported to Excel™. The research assistants, who had medical backgrounds, were well trained in the data collection process.

### Study variables and measurements

#### Dependent variable

The primary outcome was VL nonsuppression. VL suppression was defined as HIV-1 RNA VL of less than 1000 copies/mL. The variable was labeled as 0 = VL suppression and 1 = VL nonsuppression (HIV-1 RNA VL of greater than 1000 copies/mL).

#### Independent variables

The independent variables were the child’s age at ART initiation, current age, sex, ART duration, regimen, weight, WHO clinical stage, and ART adherence. The independent category variables were coded such that sex was coded as “female” or “male”; regimen was coded as “first line” or “second line”; WHO stage was coded as “stage I,” “stage II,” “stage III,” or “stage IV”; and adherence was coded as “good” or “poor.” We selected the variables based on their known clinical importance through different studies.

### Data analysis and presentation

We used Stata™ to analyze the obtained data. Descriptive statistics, such as means, standard deviations, medians, interquartile ranges (IQRs), frequencies, and percentages, are used to describe the data. Pearson chi-square tests or Fisher’s exact test were used to evaluate bivariate associations between sociodemographic variables and suppression status.

We used logistic regression to predict whether the VLS of a participant varied according to selected variables. We tested overall equality among variables in terms of log odds of VLS, and we compared the levels of the variables. We calculated predictive margins to show the probability of HVL nonsuppression using different variables, and a graph was drawn to visualize the results of the regression model.

We performed forward stepwise logistic regression, where the significance level for removal was 0.10 and the level for entry was 0.05. Adjusted odds ratios (AORs) and 95% CIs are presented.

The Hosmer and Lemeshow test was used to examine whether the final model adequately fit the data for the multiple logistic regression models. We performed an interaction test to examine the heterogeneity effect. We presented the final parsimonious model (i.e., the model with significant findings for predictors). The model-building procedure and guidelines for reporting regression analysis have previously been described in detail elsewhere [26].

## Results

### Study population characteristics

The median age of the 253 children at ART initiation was 2.0 years (IQR, 1.0–5.0 years) (Table 1), and the mean age at nonsuppression VL was 8.8 ± 2.99 years. Among the 253 children, 56% were female, and the median ART duration was 64.0 months (IQR, 37–83 months).

Most children were receiving first-line treatment regimens 99.2%; 61% had poor medication adherence; and 93% were virally suppressed (Table 1). Table 1 also shows the results of the bivariate analysis that indicate a significant variation in VLS among the medication adherence variables.

**Table 1:** Demographic and clinical characteristics of the study population according to suppression status, N = 253

Variable	Mean (std)	Median (IQR)	Suppression	Nonsuppression	Proportion N (%)	P values
Age at ART initiation (years)		2.00 (1–5)				
Current age (years)	8.8 (2.99)					
Weight at ART initiation (kg)	24.12 (7.24)					
ART duration (months		64.0 (37–83)				
Sex						
Female			134 (56.8)	9 (52.9)	143 (56.52)	0.758^*^
Male			102 (43.2)	8 (47.1)	110 (43.48)	
WHO stage^a^						
Stage I			38 (16.1)	5 (29.4)	43 (17.00)	0.577^*^
Stage II			43 (18.2)	3 (17.7)	46 (18.18)	
Stage III			116 (49.2)	7 (41.2)	123 (48.62)	
Stage IV			39 (16.5)	2 (11.8)	41 (16.21)	
Adherence						
Good			97 (41.1)	1 (5.9)	98 (38.74)	0.003^**^
Poor			139 (58.9)	16 (94.1)	155 (61.26)	
Regimen						
First-line			235 (99.6)	16 (94)	251 (99.21)	0.130^**^
Second-line			1 (0.4)	1 (5.9)	2(0.79)	

ART: Antiretroviral Therapy; ^a^WHO: World Health Organization clinical stage of HIV patients; ^*^Pearson chi-square test; ^**^ Fisher’s exact

### Predictors of VLS

Table 2 shows the results of multivariable analysis, older age at ART initiation (AOR, 1.21; 95% CI 1.012–1.443) was an independent predictor of VL nonsuppression, indicating that there is a 21% increase in the odds of VL nonsuppression for a 1-year increase in age at ART initiation. The final independent predictor of VLS was medication adherence (AOR, 0.06; 95% CI 0.004–0.867), indicating that children with high adherence had 94% lower odds of VL nonsuppression. The remaining factors examined as independent predictors of VL nonsuppression showed no significant effects.

**Table 2:** Results of multivariable analysis using multiple inputs

Variable	ODR	% change	Standard error	t	P>|z|	95% CI (lower)	95% CI (higher)
Age at ART initiation	1.21	21	0.11	2.10	0.037	1.012	1.443
Adherence							
Good	0.06	− 94	0.08	− 2.08	0.039	0.004	0.867
Poor	1					1	1
Regimen							
First-line	0.02	− 98	0.02	− 3.52	0.001	0.003	0.183
s-line	1					1	1

Visualization by regression of predictors of VLS failure

- Continuous variables

**Fig. 1:**
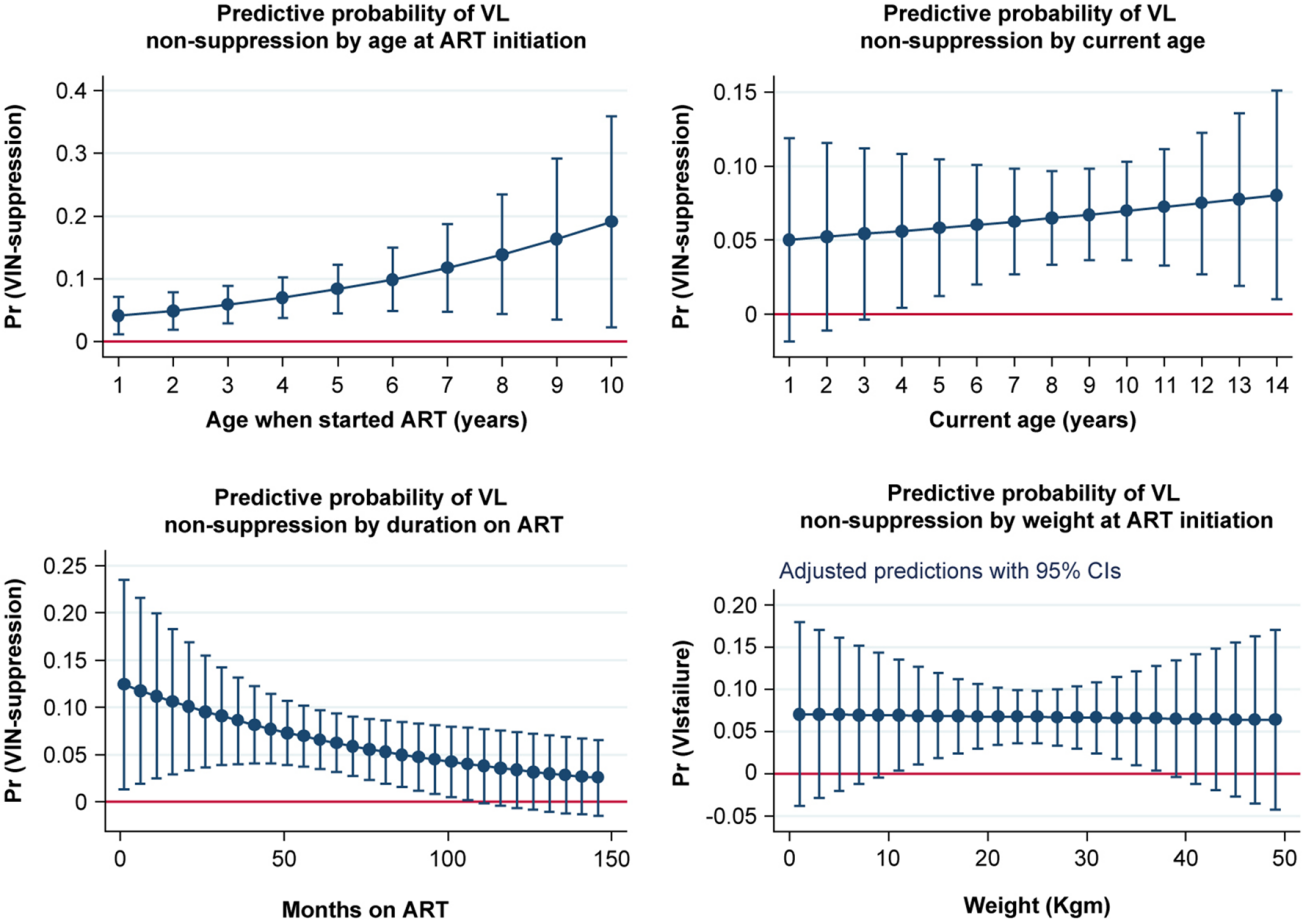
Visualization of independent continuous variables for VL nonsuppression (with 95% confidence intervals)

Figure 1 shows the relationship between the continuous independent variables and the predictive margin for the probability of HVL nonsuppression. The predictive probability of HVL nonsuppression increased as age at ART initiation increased; this relationship was significant but nonlinear (Part A). The predictive probability for VLS was also related to the age at which non VL suppression occurred. Nonsuppression trends ranged from the age of 5 years up to 14 years (Part B). Regarding ART duration, the predictive probability for VL nonsuppression decreased gradually as the ART duration increased; however, this relationship was nonlinear (Part C).

b. Categorical variables.

**Fig. 2:**
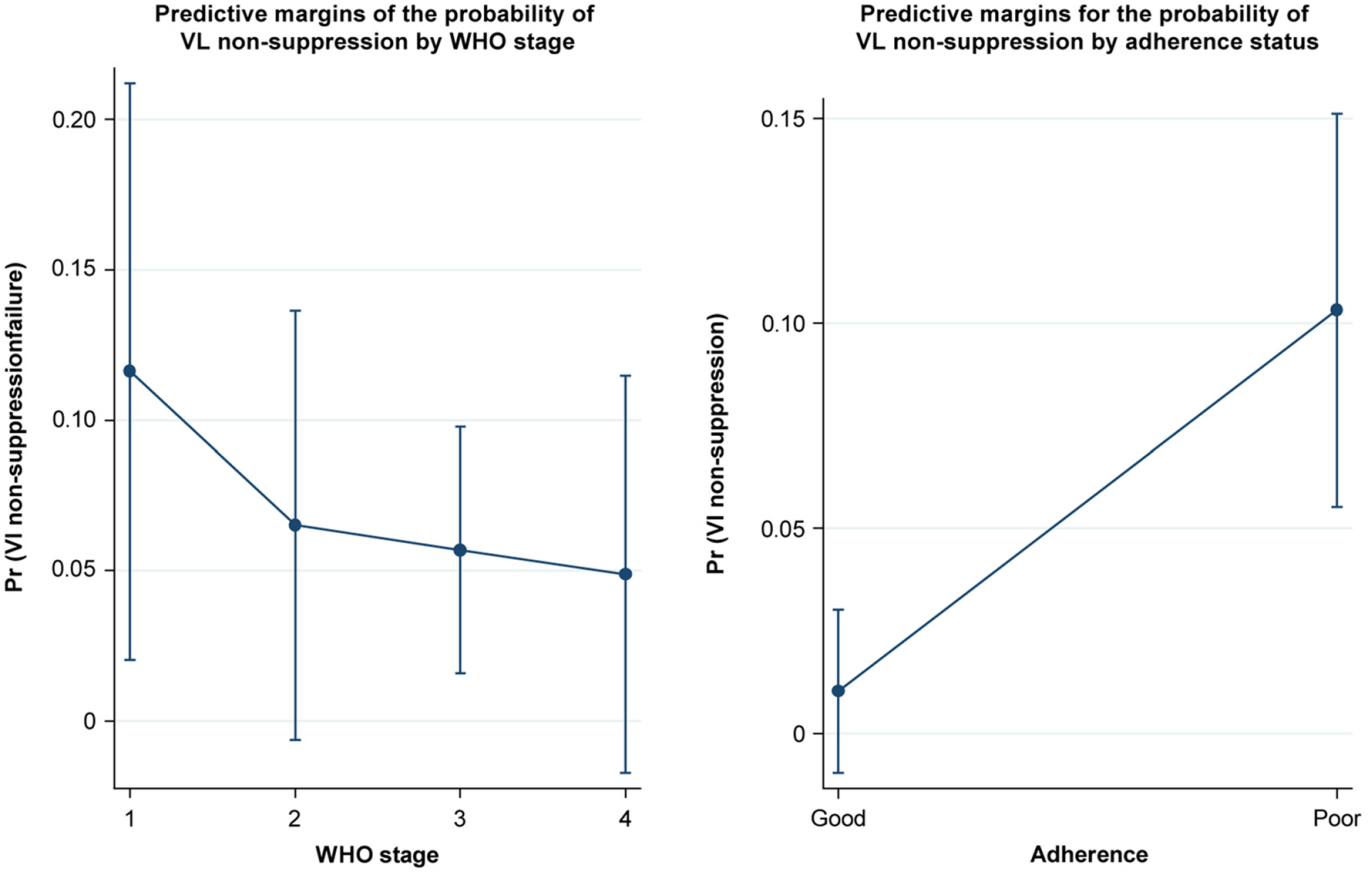
Visualization of independent categorical variables predicting viral load suppression (VLS) (adjusted prediction with 95% CIs)

Figure 2 shows the relationship between the categorical independent predictor variables and the predictive margin for the probability of HVL nonsuppression. The predictive margins for the probability of VLS by WHO stage at the time of ART initiation were nonlinear, as depicted in Part A. Part B shows that the predictive margin for VL nonsuppression was higher when it changed from good adherence to poor adherence, which was linear.

In contrast, Table 3 shows the prediction of HVL nonsuppression according to WHO stage. The log odds of VL nonsuppression for stage II were not significantly different from those for stage I (z = − 0.83, *p* = 0.406), with the same observed for stage III versus stage II (z = − 0.20, *p* = 0.839) and for stage IV versus stage III (z = − 0.20, *p* = 0.843). Thus, the WHO stage at ART initiation was not associated with VLS.

**Table 3:** Contrast among the WHO stages based on their predictive probability of HVL nonsuppression

Variable	Contrast (odds ratio)	Standard error	z	P > |z|	95% CI (lower)	95% CI (upper)
WHO stage						
(II vs. I)	0.53	0.41	− 0.83	0.406	0.12	2.37
(III vs. II)	0.87	0.62	− 0.20	0.839	0.21	3.50
(IV vs. III)	0.85	0.70	− 0.20	0.843	0.17	4.26

## Discussion

In response to the HIV epidemic, Tanzania is implementing preventive care, treatment, and support interventions to achieve the global targets of 95/95/95 by 2030 [22]. In both rural and urban settings, HIV testing and antiretroviral drugs are freely available to people with HIV. HIV VL monitoring is available and performed routinely to monitor all clients receiving ART, usually 6 months post-ART initiation.

This study revealed valuable findings regarding the factors affecting VLS in children with HIV infection who were receiving ART. In particular, approximately 17% of the interviewed participants had VL nonsuppression. Factors associated with VL nonsuppression include older age at ART initiation and poor medication adherence.

This study revealed that older age at ART initiation was significantly associated with HVL nonsuppression. Thus, HVL nonsuppression increases as age increases. The findings indicate that starting ART early, when viral replication is still low, hastens the response to therapy when compared to starting ART later, when HIV is in the advanced stage. These findings contradict each other as children get older because their adherence becomes poor and psychosocial problems become prominent after starting school. Jiamsakul et al. [10] reported that children who initiated ART at a younger age were more likely to achieve VLS. Moreover, Tagarro et al. [27] confirmed the relevance of early ART initiation as a key factor leading to smaller viral reservoir size. Children who started ART earlier were less likely to experience viral rebounds than those who starter ART later. The age at ART initiation presented a linear association with VLS, which was demonstrated by Foster et al. [28]. These findings provide additional support for the early initiation of ART in HIV-infected infants [29]. Our results indicate that early ART initiation in children with HIV reduces the risk of VLS failure; thus, interventions should focus on managing HIV in children in this age group. This result suggests that early initiation of therapy influences key virological and immunological parameters that could have significant effects on long-term health [30].

This study revealed the relationship between the ages at which VLS fails. It shows that VLS failure starts at the age of 5 years; however, this was not statistically significant and was removed from the final model. At the age of 7 years, children become partially independent because they have started school and their HIV status has been disclosed to them. Mu et al. reported on the relationship between age and VLS in children receiving ART [31]; children aged 6 years and older are more likely than younger children to have HVL nonsuppression. Older age, notably an age of more than 10 years, was associated with an increased risk of HVL nonsuppression [32]. This was potentially due to treatment nonadherence, as ART was started during a developmentally sensitive period of early life, and continuous education therapy and interventions are essential to improve adherence [33]. Our visualization indicates that age is an essential factor to consider during the care of children, particularly regarding screening and frequent follow-up, which could improve a child’s adherence to treatment.

This study revealed the relationship between ART duration and VLS. It showed that HVL nonsuppression decreased progressively as the ART duration decreased. Although this finding was not statistically significant and was removed from the final model, it could be clinically significant in the care and treatment of children. These results contradict other findings demonstrating that children who had been on ART for a longer duration (> 5 years) were more likely to achieve VLS [34], and a higher proportion of children had achieved VLS after 4 years of treatment [35]. Our visualization depicts that more intensive intervention is needed during the early months of ART initiation when nonadherence or VLS failure is detected or suspected.

This study showed that some children were switched from first-line regimens to second-line regimens due to high VL. These findings suggest the probability of early resistance of the first-line regimen to ART. Moreover, they highlighted the likelihood of resistance secondary to adherence status. Henerico et al. (2022) proved that a single high VL result (greater than 1000 copies/mL) at any point during ART is associated with a high probability of drug resistance, thus serving as a predictor of virological failure [36]. Desmond et al. [37] reported that the most common reason for switching from a first-line regimen to a second-line regimen was nonadherence, as observed in older children, who have longer periods since ART initiation. In other studies, the ART regimen type was demonstrated to be the only significant factor associated with VLS [38], whereas Johnston et al. [39] reported that nonadherence significantly contributes to regimen switching. This study did not have adequate data to demonstrate the existence of this phenomenon; thus, further studies are needed to identify the determinants of regimen changes.

This study provided evidence that medication adherence was significantly associated with VLS in this population. Children who had poor adherence were more likely to experience HVL nonsuppression than those with exemplary dedication. This shows that poor medication adherence enables the virus to replicate faster, leading to the failure of the drugs to suppress the virus. Additionally, poor adherence increases the risk of developing drug resistance and the occurrence of opportunistic infections; thus, the virus replicates more frequently and faster. Bitwale et al. showed that poor ART adherence in Tanzania was associated with virological treatment failure [40]. Similarly, another study in Tanzania showed that poor ART adherence was associated with childhood treatment failure [41]. Our findings were similar to those of other studies outside Tanzania demonstrating that nonadherence to ART increased the odds of HVL nonsuppression by fivefold [34]. Better adherence reduces the risk of VL nonsuppression [42]. These findings indicate that adherence to treatment among children receiving ART is an essential indicator of better VLS and improves well-being. Thus, HIV programs should incorporate interventions to mitigate the risk of poor adherence. However, we also observed that a substantial proportion of children who were nonadherent showed VL suppression at 6 months. This finding may be explained by the potential of most ART regimens to suppress VL even with some missed doses. Additionally, the categorization of adherence status was dichotomized where children who had missed two doses were still considered nonadherent. The categorization may have limited the ability to exhibit the linearity between the number of missed doses and the level of viral nonsuppression.

This study had several limitations. Throughout the region, the CD4 machine was not working, and the majority of children were not tested for CD4 cell count at ART initiation. Thus, there could be bias in our conclusions because these factors have significant clinical importance. The number of participants who switched from first-line regimens to second-line regimens was small. Furthermore, some children arrived with caregivers who were not their parents; therefore, they could not recall when the child became infected with HIV, so our data lack a model of participant infection. Furthermore, a cross-sectional study design was adopted in this study, which cannot show a causal relationship; thus, further analytical studies are needed, such as cohort and experimental studies. Moreover, our cross-sectional design did not include children who died or were lost to follow-up. Because these children were more likely to have experienced virologic failure, the results were biased toward better viral suppression by (naturally) excluding them.

Despite these limitations, the study’s strength was that we used advanced regression to visualize the essential factors when managing children with HIV infection.

## Conclusions

In this study, older age at ART initiation and poor medication adherence played a significant role in VLS. We recommend that interventions targeting early ART initiation for children and intensified adherence should be strengthened. Thus, there is a need to identify a sustainable intervention that promotes adherence to ART and addresses low VLS among children with HIV infection in Tanzania.

## Data Availability

Supplementary data are available in the journal and can be accessed upon request.

## Abbreviations

AOR: Adjusted odds ratio
ART: Antiretroviral therapy
CTC: Care and treatment center
HIV: Human immunodeficiency virus
IQRs: Interquartile ranges
PLHIV: People living with HIV
VL: Viral load
VLS: Viral load suppression
